# Clinical and Prognostic Significance of Positive Hepatojugular Reflux on Discharge in Acute Heart Failure: Insights from the ESCAPE Trial

**DOI:** 10.1155/2017/5734749

**Published:** 2017-02-21

**Authors:** Hesham R. Omar, Maya Guglin

**Affiliations:** ^1^Internal Medicine Department, Mercy Medical Center, Clinton, IA, USA; ^2^Division of Cardiovascular Medicine, Linda and Jack Gill Heart Institute, University of Kentucky, Lexington, KY, USA

## Abstract

*Background*. There has been a decline in emphasis of the value of physical examination in heart failure (HF) with increased reliance on cardiac imaging. We aim to study the clinical and prognostic significance of positive hepatojugular reflux (HJR) on discharge in patients hospitalized with HF.* Methods*. Using the ESCAPE trial data, patients were compared according to the presence or absence of a positive HJR on discharge. The primary study endpoints were all-cause mortality and a composite endpoint of death, rehospitalization, and cardiac transplant during the first 6 months after discharge.* Results*. Among 392 patients (age: 56 years, 74% men), the HJR correlated well with clinical and objective hemodynamic markers of volume overload including right atrial pressure (RAP, *P* = 0.002), pulmonary capillary wedge pressure (PCWP, *P* = 0.006), and inferior vena cava size during inspiration (*P* = 0.005) and expiration (*P* = 0.003). The RAP had the highest AUC for predicting a positive HJR on admission (AUC: 0.655, *P* = 0.004) and discharge (AUC: 0.672, *P* = 0.001). Cox's proportional hazards analysis revealed that a positive HJR on discharge is an independent predictor of 6-month mortality (estimated hazard ratio: 1.689; 95% CI: 1.032–2.764; *P* = 0.037) after adjusting for age, baseline creatinine, baseline hematocrit, baseline NYHA class, chronic obstructive pulmonary disease, and the presence of tricuspid regurgitation.* Conclusion*. The HJR should be routinely checked in patients admitted with acute HF throughout hospitalization and especially on discharge as it serves as an important prognostic marker for postdischarge outcomes.

## 1. Introduction

Heart failure (HF) affects 26 million patients worldwide causing 1 million hospitalization cases annually in the United States [1] and accounting for a total Medicare expenditure exceeding $17 billion [2]. Those patients hospitalized with HF continue to experience high mortality and readmission rate which is greater than 50% within 6 months after discharge [3–5] despite achievements in medical and device therapy. Volume overload is the commonest reason for hospitalization for HF [6–8] and is evident in some HF patients on discharge despite appropriate in-hospital treatment [6]. Hepatojugular reflux (HJR) is one of the clinical signs of volume overload, easily obtainable at bedside.

The HJR is a simple, reliable, but neglected physical exam sign useful for diagnosing and managing HF. A positive HJR sign is defined by an increase in the jugular venous pressure (JVP) > 3 cm, sustained for greater than 15 seconds, and signifies that the right ventricle cannot accommodate the augmented venous return. It is commonly evident in left ventricular failure if the pulmonary capillary wedge pressure (PCWP) is greater than 15 mmHg. In the absence of left HF, a positive HJR should prompt evaluation for pathologies affecting right ventricular preload, afterload, compliance, or systolic function. This sign is especially important in early HF with modest volume increase before conventional symptoms and signs become evident. In this instance, the measurement of the JVP may not be the best tool to assess the volume status. The HJR was originally described by Pasteur as a manifestation of tricuspid regurgitation (TR) [9]. However, a positive HJR was later found to be present in congestive HF irrespective of its etiology. It has high specificity in diagnosing HF [10] and predicting elevated right atrial pressure (RAP) [11].

Since the initial publication of the original report by Pasteur in 1885, there is only minimal research regarding the significance of a positive HJR in HF highlighting the degree of neglect and underuse of this important physical sign. The association between a positive HJR on discharge in patients admitted to the hospital with acute decompensated HF and various postdischarge outcomes has not been previously explored. In this analysis, we study the determinants, clinical significance, and prognostic implication of the presence of a positive HJR in patients with acute systolic HF.

## 2. Methods

This study is a retrospective analysis of a limited access dataset from the original Evaluation Study of Congestive Heart Failure and Pulmonary Artery Catheterization Effectiveness (ESCAPE) trial provided by the National Heart, Lung, and Blood Institute (NHLBI). According to the NHLBI policy, datasets from major trials include the protocol and all collected variables with their definitions. All personal identifiers are excluded.

The ESCAPE trial compared outcomes of patients with acute HF managed with clinical assessment plus pulmonary artery catheterization (PAC) versus clinical assessment alone. The study enrolled 433 patients with severe symptomatic HF using the following inclusion criteria: (1) hospitalization for decompensated HF within the past year, (2) urgent visit to the emergency department, and (3) treatment during the prior month with 160 mg of furosemide or its equivalent. In addition, randomization required patients to have at least 3 months of symptoms despite being on angiotensin-converting enzyme inhibitors and diuretics, systolic blood pressure ≤125 mm Hg, and left ventricular ejection fraction ≤30% in addition to at least one symptom and one sign of congestion. Results of the ESCAPE trial have been previously published [12].

All patients had detailed physical examination variables checked from the time of admission through 6 months after discharge. Among the physical examination variables is the HJR. We have included in this analysis all patients who had an assessment of the HJR on hospital discharge (*n* = 392). These cases were divided into two groups based on the presence or absence of a positive HJR. The primary study endpoints were all-cause mortality and a composite endpoint of death, rehospitalization, and cardiac transplant during the first 6 months after hospital discharge. We also aimed to find the determinants of a positive HJR and to evaluate the reliability of this clinical sign through studying its association with other clinical as well as objective hemodynamic variables of congestion measured by the PAC and echocardiography.

### 2.1. Statistical Analysis

Primary analysis compared patients with and without a positive HJR on hospital discharge. Continuous variables were tested for normality of distribution using the Shapiro-Wilk test. They were expressed as mean ± standard deviation and compared using independent samples *t*-test for normally distributed variables and the Mann–Whitney test for non-normally distributed variables. Categorical variables are described as counts and percentages and compared using the Chi-square test. Univariate odds ratio (OR) was determined using the Cochran-Mantel-Haenszel common odds ratio estimate. A receiver operating characteristics (ROC) analysis was implemented to detect the best determinant of a positive HJR and to calculate the area under curve (AUC). In order to examine the relationship between HJR on discharge and all-cause mortality at 6 months, Cox's proportional hazards model was used. The variables used in these models were either pertinent variables that achieved statistical significance at *P* < 0.01 upon univariate analysis or those considered to be clinically relevant. Comparisons of time-to-death outcome between patients with or without HJR on discharge are shown using Kaplan-Meier estimates and log-rank tests. A *P* value less than 0.05 was considered statistically significant. All statistical significance was assessed using 2-sided *P* values. Data were analyzed using IBM SPSS 21.0 statistical software (IBM SPSS Version 21.0., Armonk, NY).

## 3. Results

### 3.1. Baseline Characteristics

A total of 392 patients (mean age: 56 years, 74% men) with available information about HJR on discharge were included in the analysis. 86% of the patients were classified as New York Heart Association (NYHA) class IV and 14% were class III HF, and the mean hospital stay was 8.5 days. 227 patients (58%) were rehospitalized, out of which 179 (45.7%) were rehospitalized for HF. 71 of the 392 patients (18.1%) died during the study period, out of which 23 patients (5.9%) died in hospital. Of the 392 patients who had data on HJR on discharge, 115 (29.3%) had a positive HJR and 277 (70.7%) had a negative HJR. 192/392 (49%) patients were treated with guidance of a PAC. Compared with those with a negative HJR on discharge, patients with a positive HJR on discharge were older (*P* = 0.003) and had a higher frequency of NYHA class IV symptoms at baseline (*P* = 0.034) and discharge (*P* = 0.023) and higher frequency of ischemic heart disease (*P* = 0.011), tricuspid regurgitation (TR, *P* = 0.02), and higher creatinine at baseline (*P* = 0.039). Comparison of patients with or without a positive HJR on discharge is shown in Table 1.

### 3.2. Association of HJR with Clinical, Laboratory, and Objective Markers of Congestion

With regard to discharge physical examination, patients with a positive HJR on discharge had a higher frequency of JVP > 8 cm (*P* < 0.001) and higher frequency of at least moderate ascites (*P* = 0.001), hepatomegaly (*P* < 0.001), and rales (*P* = 0.001). With regard to discharge laboratory variables, patients with a positive HJR had a higher discharge B-type natriuretic peptide (BNP, *P* = 0.002) and blood urea nitrogen level (*P* = 0.019) and lower hematocrit (*P* = 0.026). With regard to hemodynamic parameters of overload, patients with positive HJR on discharge had a higher RAP (*P* = 0.002), pulmonary artery systolic pressure (PASP, *P* = 0.005), pulmonary artery diastolic pressure (*P* = 0.009), mean pulmonary artery pressure (*P* = 0.01), and PCWP (*P* = 0.006), all measured by PAC on the last day of hemodynamic measurement. Evaluation of discharge echocardiographic data showed that a positive HJR was associated with larger inferior vena cava (IVC) size during inspiration (*P* = 0.005) and expiration (*P* = 0.003).  Table 2 shows the relationship between positive HJR on discharge and clinical, laboratory, echocardiographic, and central hemodynamic variables of congestion.

### 3.3. Determinants of a Positive HJR

To identify the best hemodynamic variable that determines a positive HJR on discharge, we performed a ROC curve analysis. The RAP had the highest AUC in predicting a positive HJR on discharge (0.672; 95% CI: 0.566–0.779; *P* = 0.001). The PASP had an AUC of 0.663 (95% CI: 0.560–0.765; *P* = 0.003), PCWP had an AUC of 0.646 (95% CI: 0.541–0.751; *P* = 0.007), and the PADP had an AUC of 0.607 (95% CI: 0.503–0.712; *P* = 0.047). The RAP was also the main predictor of a positive HJR across other study time points. On admission, the RAP had the highest AUC in predicting a positive HJR (0.655; 95% CI: 0.551–0.760; *P* = 0.004). Comparison of the area under ROC curves on admission revealed significantly higher AUC of RAP compared with PADP (*P* = 0.0317) and RAP compared with PCWP (*P* = 0.0373) in predicting a positive HJR.

### 3.4. Univariate Relationship between Positive HJR on Discharge and Outcomes

On univariate analysis, a positive HJR on discharge was associated with higher 6-month mortality (27% versus 14.4% in those with and without positive HJR on discharge, resp.; univariate OR: 2.187; 95% CI: 1.286–3.718; *P* = 0.004). In addition, mortality was also consistently higher in those with a positive HJR across multiple study time points including day 3 (24.4% versus 14.2%, *P* = 0.016), day 5 (32.3% versus 18%, *P* = 0.012), week 2 (23.5% versus 11.9%, *P* = 0.006), 1 month (19% versus 7.8%, *P* = 0.004), and 3 months (13.9% versus 3.3%, *P* = 0.002). A positive HJR on discharge was also associated with a higher frequency of composite endpoint of death, rehospitalization, and cardiac transplant (71.3% versus 60.3%, resp.; univariate OR: 1.637; 95% CI: 1.023–2.62; *P* = 0.04) which was also consistently higher in those with a positive HJR across multiple study time points including day 3 (73.9% versus 58.5%, *P* = 0.002), day 7 (90.7% versus 70.1%, *P* = 0.006), and 6 months (65.2% versus 46.8%, *P* = 0.013). Univariate relationships between positive HJR on discharge and postdischarge outcomes are listed in Table 3.

### 3.5. Longitudinal Follow-Up of the HJR

In order to examine the importance of longitudinal follow-up of the HJR during hospitalization, we studied outcomes in those with a positive HJR on admission who had either resolution of HJR or persistent HJR on the day of discharge. We found that, among 303 patients with a positive HJR on admission, 108 had persistent HJR on discharge, while 195 had resolution of HJR on discharge. Patients who had persistent HJR on discharge had a higher risk of 6-month mortality compared with those who had resolution of HJR on discharge (univariate OR: 2.167; 95% CI: 1.189–3.949; *P* = 0.012).

### 3.6. Value of Combined Assessment of HJR and JVD

We have evaluated the role of combined assessment of positive HJR and jugular venous distension (JVD) in postdischarge mortality. We compared patients with positive HJR and JVD (*n* = 65) on discharge with patients with positive HJR and no JVD (*n* = 48). JVD was defined if JVP was >8 mmHg. Patients with positive HJR and JVD on discharge had higher 6-month mortality compared with those with positive HJR and no JVD (33.8% versus 16.7%, resp.; univariate OR: 2.558; 95% CI: 1.023–6.397; *P* = 0.045).

### 3.7. Multivariate Analysis to Identify Independent Predictors of 6-Month Mortality

**Figure 1:**
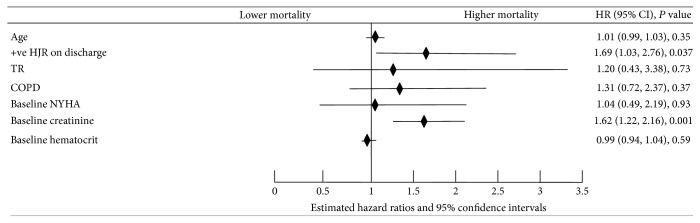
Forest plot showing results of Cox's proportional hazards analysis of 6-month mortality for ESCAPE trial patients admitted with acute systolic heart failure. HJR: hepatojugular reflux; TR: tricuspid regurgitation; COPD: chronic obstructive pulmonary disease; NYHA: New York Heart Association class.

**Figure 2:**
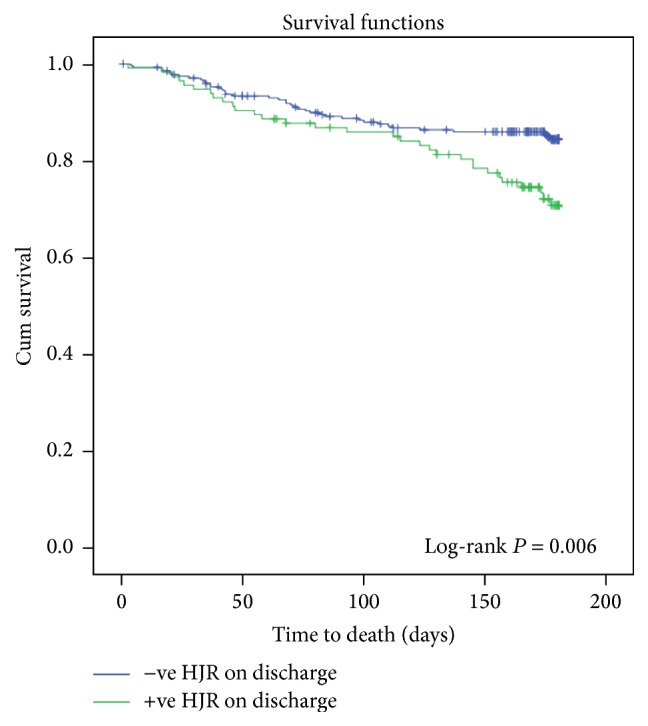
Kaplan-Meier cumulative survival curves in ESCAPE trial patients hospitalized with acute systolic heart failure showing a significant difference in survival between those who have either positive or negative hepatojugular reflux on discharge.

A total of 71/392 (18.1%) patients died at 6 months. Cox's proportional hazard analysis revealed that a positive HJR on discharge is an independent predictor of 6-month mortality (estimated hazards ratio: 1.689; 95% CI: 1.032–2.764; *P* = 0.037) after adjusting for known postdischarge mortality predictors in HF including age, baseline creatinine, baseline NYHA classification, chronic obstructive pulmonary disease, hematocrit at baseline, and the presence of TR.  Figure 1 is a forest plot showing a summary of variables used in the multivariate model.  Figure 2 is a Kaplan-Meier survival curve comparing survival in patients with or without a positive HJR on discharge (log-rank *P* value = 0.006).

## 4. Discussion

We have shown in this study the importance of evaluating the HJR in patients hospitalized with acute HF. The HJR is a simple bedside clinical sign with high intraobserver agreement of ~97% [13], which correlated well with physical exam signs of congestion and BNP as well as central hemodynamic parameters of volume overload: the PCWP and RAP. This association was unaltered even after controlling for the presence of TR suggesting that volume overload is a contributing factor. The HJR is considered by many as an “eye into the heart” that is a good, inexpensive, and noninvasive alternative for hemodynamic monitoring in HF. Prior studies showed that the HJR was a useful tool in predicting HF in patients presenting with dyspnea [14] and was associated with a PCWP ≥ 15 mmHg [15]. Prior studies also found high specificity for the HJR (~96%) in diagnosing HF [15, 16]. In addition to confirming these prior findings, we have also found that a positive HJR on discharge was determined by higher RAP, PASP, and PADP and was associated with a higher IVC diameter which is an accurate determinant of patients' volume status with ability to predict decompensated HF [17]. While studies showed that HJR correlated best with left-sided HF [15] and others showed stronger association with right-sided pressures [11], we found a significant relationship between a positive HJR and RAP, PCWP, PASP, and PADP, suggesting that it is a marker of elevated left-sided or right-sided filling pressures. Because the timing of the HJR on discharge did not accurately coincide with RAP, PASP, PADP, and PCWP, which were rather recorded on the last day of hemodynamic measurement and not necessarily the day of discharge, we have compared area under ROC curve of various hemodynamic measurements on admission in predicting a positive HJR on the day of hospitalization and found that the AUC of RAP was significantly more than PADP (*P* = 0.0317) and PCWP (*P* = 0.0373).

Despite the significant advances in HF therapy, many patients are discharged with signs and symptoms of volume overload [6]. It was found that patients rehospitalized with HF were not optimally decongested at time of discharge from prior hospitalization. Persistent congestion is associated with increased HF rehospitalization and death [18–21] and this risk increases with repeated hospitalization. In a recent analysis of the ESCAPE trial, Cooper and colleagues found that postdischarge outcomes of patients with HF were mainly driven by persistent congestion (higher right- and left-sided filling pressures) but not the cardiac index [22]. Our study confirms the findings of the effect of congestion on postdischarge mortality and composite endpoint of death, rehospitalization, and cardiac transplant. In our analysis, patients with a positive HJR on discharge were 1.7 times (*P* = 0.03) at risk of death at 6 months. This independent association of a positive HJR on discharge and mortality is likely because the HJR is a proxy for persistent congestion due to suboptimal treatment of acute HF. This was evident in the fact that patients with a positive HJR had higher frequency of clinical signs of congestion on discharge including rales (*P* = 0.001), at least moderate ascites (*P* = 0.001), hepatomegaly (*P* < 0.001), and JVP > 8 cm (*P* < 0.001). The effects of congestion on various body organs in HF likely explain the association between positive HJR and mortality. For example, it was shown that worse renal function in acute HF is related more to the degree of congestion rather than decreased forward flow and is associated with worse outcomes [23]. Another explanation for the association of a positive HJR on discharge and mortality is the possibility that patients with positive HJR may have worse HF pathology. We have also demonstrated the importance of longitudinal follow-up of the HJR from admission to discharge, where those admitted with a positive HJR that was persistent on discharge had higher risk of death compared to those who had resolution of the HJR on discharge (OR: 2.167; *P* = 0.012).

In the United States, there is a gradual decline in emphasis of the value of physical examination [24, 25], with increased reliance on laboratory and imaging studies. This reflects an erroneous perception that the physical examination provides limited information [26] compared with modern diagnostic tools. In fact, a previous analysis from the ESCAPE trial to investigate the value of the history and physical examination showed that, in advanced HF, the presence of orthopnea and increased JVP are useful to detect increased PCWP; in addition, hemodynamic profiles obtained from the discharge physical examination identified patients at increased risk of early events [27]. Reliance on physical examination signs in HF is especially of importance in low-income countries, where clinical examination remains the cornerstone of diagnosis and management due to the minimal financial support dedicated for healthcare allowing the limited use of imaging like echocardiography or even a simple chest X-ray or BNP testing.

## 5. Study Limitations

There are several limitations for this study. The analysis was retrospective. Although all centers enrolled in the ESCAPE trial were experienced in the management of advanced HF, the HJR is still a subjective physical finding and its validity and reliability will not be 100%. The defining criterion for a positive HJR was not outlined in the study. Also, the severity of TR was not routinely assessed and therefore we cannot study its impact on the RAP and subsequently the HJR. However, we have controlled for the presence of TR in multivariable analysis. The various treatment strategies were not identified in our limited dataset. Because this was a multicenter trial, patients with similar hemodynamic profiles at different centers may have received different treatments which may have acted as potential confounders and affected the outcomes. There may have been other confounders that have not been accounted for and affected mortality like noncardiac comorbidities (e.g., pneumonia and sepsis). Because the ESCAPE trial included very sick patients with acute systolic HF, this value of HJR may not be applicable in patients with chronic HF. Also, unlike acute HF, it is known that physical signs of congestion in patients with chronic HF (e.g., rales, edema, and jugular venous distension) have low sensitivity in predicting volume overload [28]; therefore our findings are limited to acute HF.

## 6. Conclusion

The presence of a positive HJR on discharge of patients hospitalized with decompensated systolic HF correlates well with objective markers of volume overload and is an independent predictor of 6-month mortality. The HJR should be routinely checked in patients admitted with acute HF throughout hospitalization and especially on discharge as it serves as an important prognostic tool for postdischarge outcomes.

## Figures and Tables

**Table 1:** Demographic, clinical, laboratory, hemodynamic, and echocardiographic characteristics of patients with or without hepatojugular reflux on discharge enrolled in the ESCAPE trial.

	+ve HJR on discharge (*n* = 115)	−ve HJR on discharge (*n* = 277)	*P* value
*Baseline demographics*			
Age (years, m ± SD)	59.3 ± 13.6	54.7 ± 13.8	0.003
Male sex % (*n*)	71.3% (82/115)	74.7% (207/277)	0.483
White race % (*n*)	60.9% (70/115)	59.9% (166/277)	0.862
BMI on admission (Kg/m^2^, m ± SD)	27.9 ± 6.4	29.1 ± 6.9	0.119
BMI on discharge (Kg/m^2^, m ± SD)	26.8 ± 6	27.9 ± 6.9	0.199

*Comorbidities*			
Ischemic heart disease % (*n*)	63.7% (72/113)	49.5% (137/277)	0.011
Atrial fibrillation % (*n*)	36.3% (41/113)	26.7% (74/277)	0.061
CABG % (*n*)	34.5% (39/113)	27.1% (75/277)	0.144
Stroke % (*n*)	8% (9/113)	9.7% (27/277)	0.582
Hypertension % (*n*)	49.6% (56/113)	45.1% (125/277)	0.426
Hepatic disease % (*n*)	10.6% (12/113)	7.6% (21/277)	0.330
DM on oral medication % (*n*)	16.8% (19/113)	17% (47/277)	0.971
COPD % (*n*)	19.5% (22/113)	15.5% (43/277)	0.344
PVD % (*n*)	20.4% (23/113)	9% (25/277)	0.003
Mitral regurgitation % (*n*)	11.5% (13/113)	10.1% (28/277)	0.684
Tricuspid regurgitation % (*n*)	8% (9/113)	2.5% (7/277)	0.02
NYHA class IV at baseline % (*n*)	92% (104/113)	83.4% (231/277)	0.034

*Clinical and hemodynamic variables on discharge*			
Supine SBP (mmHg, m ± SD)	102.5 ± 15.4	101.2 ± 14.3	0.413
Supine DBP (mmHg, m ± SD)	61.5 ± 10.8	61.3 ± 10.9	0.925
Supine heart rate (bpm, m ± SD)	79.7 ± 14.3	79.5 ± 13.9	0.918
Respiratory rate (breath/min, m ± SD)	18.6 ± 3.4	18.6 ± 2.4	0.234
6-Minute walk distance (feet, m ± SD)	823 ± 403	794 ± 356	0.734
NYHA class IV at discharge % (*n*)	23.9% (27/113)	14.2% (39/274)	0.023

*Laboratory variables on discharge*			
BUN (mg/dL, m ± SD)	42.2 ± 25	35.3 ± 19.7	0.019
Creatinine (mg/dL, m ± SD)	1.6 ± 0.7	1.5 ± 0.8	0.068
Total bilirubin (mg/dL, m ± SD)	0.83 ± 0.48	0.85 ± 0.48	0.809
Direct bilirubin (mg/dL, m ± SD)	0.39 ± 0.37	0.36 ± 0.32	0.996
AST (U/L, m ± SD)	39.7 ± 51.1	34.5 ± 23	0.661
ALT (U/L, m ± SD)	43.7 ± 101.6	33.7 ± 32.7	0.701
Na (meq/L, m ± SD)	135 ± 5.1	135.4 ± 4	0.758
Hematocrit (%, m ± SD)	36.9 ± 5.8	42.4 ± 40.9	0.026
Troponin I (ng/mL, m ± SD)	0.03 ± 0.02	0.15 ± 0.39	0.753

*Echocardiographic data on discharge*			
TR velocity (m ± SD)	3.1 ± 0.5	2.9 ± 0.5	0.04
EF (m ± SD)	22.4 ± 10	20.2 ± 8.2	0.189
RA area (m ± SD)	27 ± 8.3	25.1 ± 8.2	0.142
RV area in systole (m ± SD)	19.6 ± 7	18.3 ± 7.2	0.236
RV area in diastole (m ± SD)	25.8 ± 7.4	24.4 ± 7.6	0.251
E/A ratio (m ± SD)	2.68 ± 1.46	2.66 ± 4.83	0.009
Deceleration of *E* velocity (m ± SD)	135 ± 33	157 ± 69	0.261

BMI: body mass index; CABG: coronary artery bypass graft; DM: diabetes mellitus; COPD: chronic obstructive pulmonary disease; PVD: peripheral vascular disease; NYHA: New York Heart Association; SBP: systolic blood pressure; DBP: diastolic blood pressure; BUN: blood urea nitrogen; EF: ejection fraction; RV: right ventricle.

**Table 2:** Comparison of clinical, laboratory, echocardiographic, and central hemodynamic variables of congestion among patients enrolled in the ESCAPE trial who have positive or negative hepatojugular reflux on discharge.

	+ve HJR on discharge (*n* = 115)	−ve HJR on discharge (*n* = 277)	*P* value
*Clinical and laboratory variables of congestion*			
JVD at discharge >8 cm % (*n*)	57.5% (65/113)	25.9% (68/263)	<0.001
At least moderate ascites % (*n*)	9.6% (11/115)	1.1% (3/276)	0.001
Hepatomegaly % (*n*)	40.9% (47/115)	8.9% (24/271)	<0.001
Rales % (*n*)	16.5% (19/115)	5.8% (16/277)	0.001
BNP (pg/mL, m ± SD)	936 ± 1428	659 ± 1041	0.002

*Echocardiography variables of congestion*			
IVC size in inspiration (cm, m ± SD)	1.5 ± 0.73	1.1 ± 0.78	0.005
IVC size in expiration (cm, m ± SD)	2.1 ± 0.67	1.8 ± 0.7	0.003

*PAC data at final hemodynamic measurement*			
RAP (mmHg, m ± SD)	11.1 ± 6.5	7.8 ± 4.2	0.002
PASP (mmHg, m ± SD)	49.9 ± 13	43.9 ± 11.6	0.005
PADP (mmHg, m ± SD)	23.2 ± 8.2	20 ± 6.2	0.009
PAMP (mmHg, m ± SD)	32.3 ± 11.5	28.3 ± 7.8	0.01
PCWP (mmHg, m ± SD)	19.8 ± 8	16.1 ± 6	0.006

HJR: hepatojugular reflux; JVD: jugular venous distension; BNP: B-type natriuretic peptide; IVC: inferior vena cava; RAP: right atrial pressure; PASP: pulmonary artery systolic pressure; PADP: pulmonary artery diastolic pressure; PAMP: pulmonary artery mean pressure; PCWP: pulmonary capillary wedge pressure.

**Table 3:** Short-term and intermediate-term outcomes of patients with or without a positive hepatojugular reflux on discharge enrolled in the ESCAPE trial.

	+ve HJR on discharge (*n* = 115)	−ve HJR on discharge (*n* = 277)	*P* value
Mortality % (*n*)	27% (31/115)	14.4% (40/277)	0.004
Composite endpoint of death, rehospitalization, and cardiac transplant % (*n*)	71.3% (82/115)	60.3% (167/277)	0.04
Death due to witnessed cardiac arrest % (*n*)	58.3% (7/12)	13.3% (2/15)	0.021
Number of days of initial hospitalization (days, m ± SD)	8.8 ± 7.4	7.8 ± 5.6	0.228
Total days in hospital in first 180 days	17.6 ± 17.9	16.1 ± 18.7	0.208
Patient received LVAD or cardiac transplant % (*n*)	5.2% (6/115)	8.7% (24/277)	0.247

HJR: hepatojugular reflux; LVAD: left ventricular assist device.

## Abbreviations

HF: Heart failure
HJR: Hepatojugular reflux
RAP: Right atrial pressure
TR: Tricuspid regurgitation
OR: Odds ratio
ROC: Receiver operator characteristic curve
AUC: Area under curve
NYHA: New York Heart Association
PASP: Pulmonary artery systolic pressure
PCWP: Pulmonary capillary wedge pressure
IVC: Inferior vena cava
BMI: Body mass index
LVAD: Left ventricular assist device
ESCAPE: Evaluation Study of Congestive Heart Failure and Pulmonary Artery Catheterization Effectiveness.

## References

[1] Ambrosy A. P., Fonarow G. C., Butler J. (2014). The global health and economic burden of hospitalizations for heart failure. *Journal of the American College of Cardiology*.

[2] Rosamond W., Flegal K., Furie K. (2008). Heart disease and stroke statistics—2008 Update: a report from the American heart association statistics committee and stroke statistics subcommittee. *Circulation*.

[3] Krumholz H. M., Merrill A. R., Schone E. M. (2009). Patterns of hospital performance in acute myocardial infarction and heart failure 30-day mortality and readmission. *Circulation: Cardiovascular Quality and Outcomes*.

[4] Joynt K. E., Jha A. K. (2011). Who has higher readmission rates for heart failure, and why? implications for efforts to improve care using financial incentives. *Circulation: Cardiovascular Quality and Outcomes*.

[5] Chun S., Tu J. V., Wijeysundera H. C. (2012). Lifetime analysis of hospitalizations and survival of patients newly admitted with heart failure. *Circulation: Heart Failure*.

[6] O'Connor C. M., Stough W. G., Gallup D. S., Hasselblad V., Gheorghiade M. (2005). Demographics, clinical characteristics, and outcomes of patients hospitalized for decompensated heart failure: observations from the IMPACT-HF registry. *Journal of Cardiac Failure*.

[7] Gheorghiade M., Filippatos G., De Luca L., Burnett J. (2006). Congestion in acute heart failure syndromes: an essential target of evaluation and treatment. *American Journal of Medicine*.

[8] Cleland J. G. F., Swedberg K., Follath F. (2003). The EuroHeart Failure survey programme—a survey on the quality of care among patients with heart failure in Europe: part 1: patient characteristics and diagnosis. *European Heart Journal*.

[9] Pasteur W. (1885). Note on a new physical sign of tricuspid regurgitation. *The Lancet*.

[10] Maisel A. S., Atwood J. E., Goldberger A. L. (1984). Hepatojugular reflux: useful in the bedside diagnosis of tricuspid regurgitation. *Annals of Internal Medicine*.

[11] Sochowski R. A., Dubbin J. D., Naqvi S. Z. (1990). Clinical and hemodynamic assessment of the hepatojugular reflux. *The American Journal of Cardiology*.

[12] Binanay C., Califf R. M., Hasselblad V. (2005). Evaluation study of congestive heart failure and pulmonary artery catheterization effectiveness: the ESCAPE trial. *The Journal of the American Medical Association*.

[13] Butman S. M., Ewy G. A., Standen J. R., Kern K. B., Hahn E. (1993). Bedside cardiovascular examination in patients with severe chronic heart failure: importance of rest or inducible jugular venous distension. *Journal of the American College of Cardiology*.

[14] Wiese J. (2000). The abdominojugular reflux sign. *American Journal of Medicine*.

[15] Ewy G. A. (1988). The abdominojugular test: technique and hemodynamic correlates. *Annals of Internal Medicine*.

[16] Marantz P. R., Kaplan M. C., Alderman M. H. (1990). Clinical diagnosis of congestive heart failure in patients with acute dyspnea. *Chest*.

[17] Besli F., Kecebas M., Caliskan S., Dereli S., Baran I., Turker Y. (2015). The utility of inferior vena cava diameter and the degree of inspiratory collapse in patients with systolic heart failure. *American Journal of Emergency Medicine*.

[18] Drazner M. H., Rame J. E., Stevenson L. W., Dries D. L. (2001). Prognostic importance of elevated jugular venous pressure and a third heart sound in patients with heart failure. *New England Journal of Medicine*.

[19] Sachdeva A., Horwich T. B., Fonarow G. C. (2010). Comparison of usefulness of each of five predictors of mortality and urgent transplantation in patients with advanced heart failure. *American Journal of Cardiology*.

[20] Fonarow G. C., Stevenson L. W., Steimle A. E. (1994). Persistently high left-ventricular filling pressures predict mortality despite angiotensin-converting enzyme-inhibition in advanced heart failure. *Circulation*.

[21] Fonarow G., Hamilton M., Moriguchi J., Creaser J., Rourke D. A. (2001). Hemodynamic predictors of clinical outcomes in decompensated advanced heart failure. *Journal of Cardiac Failure*.

[22] Cooper L. B., Mentz R. J., Stevens S. R. (2016). Hemodynamic predictors of heart failure morbidity and mortality: fluid or flow?. *Journal of Cardiac Failure*.

[23] Metra M., Davison B., Bettari L. (2012). Is worsening renal function an 338 ominous prognostic sign in patients with acute heart failure? The role of congestion and its 339 interaction with renal function. *Circulation: Heart Failure*.

[24] Mangione S. (2001). Cardiac auscultatory skills of physicians-in-training: a comparison of three english-speaking countries. *American Journal of Medicine*.

[25] Mangione S., Nieman L. Z. (1997). Cardiac auscultatory skills of internal medicine and family practice trainees: a comparison of diagnostic proficiency. *JAMA*.

[26] Rame J. E., Dries D. L., Drazner M. H. (2003). The prognostic value of the physical examination in patients with chronic heart failure. *Congestive Heart Failure*.

[27] Drazner M. H., Hellkamp A. S., Leier C. V. (2008). Value of clinician assessment of hemodynamics in advanced heart failure: the ESCAPE trial. *Circulation. Heart failure*.

[28] Leier C. V., Chatterjee K. (2007). The physical examination in heart failure—Part II. *Congestive Heart Failure*.

